# Cabrol technique in coronary artery bypass grafting

**DOI:** 10.1186/1749-8090-10-S1-A290

**Published:** 2015-12-16

**Authors:** Chan-Young Na, Tae-Shik Kim

**Affiliations:** 1 Department of Thoracic & Cardiovascular Surgery, Keimyung University, Daegu, 41931, South Korea; 2 Department of Thoracic & Cardiovascular Surgery, Korea University, Seoul, 02841, South Korea

## Background/Introduction

In coronary artery bypass surgery, proximal anastomosis between the ascending aorta and an arterial or venous graft may be conducted by side-to-side maneuver (Cabrol-type).

## Aims/Objectives

We evaluated the long-term clinical outcome and aortocoronary graft patency of Cabrol-type proximal anastomosis in coronary artery bypass grafting (CABG).

## Method

From 2002 to 2012, 460 patients (age, 64.7 ± 8.3 years) underwent CABG using Cabrol technique. The graft configuration included the anastomosis of saphenous vein (SV) to saphenous vein (n = 266), SV to radial artery (RA) (n = 65), RA to SV (n = 108), RA to RA (n = 8), and others (n = 11) (Figure). The mean follow-up duration was 50.3 ± 32.3 months. Postoperatively, coronary computed tomography angiography (CTA) was checked in 362 patients (78.7%).

## Results

The operative mortality was 3.9%. The actuarial rate of the overall survival at 1, 5, and 10 years was 97.7% ± 0.7%, 88.6% ± 1.6%, and 70.4% ± 4.0%, respectively. The actuarial MACCE-free survival at 1, 5, and 10 years was 97.7% ± 0.7%, 89.9% ± 1.6%, and 84.2% ± 2.8%, respectively. Of 301 patients who used LITA (in situ) to LAD anastomosis, 712 grafts (mean, 2.4 grafts per patient) were used in Cabrol-type anastomosis. The 1-, 2-, 5-, and 8-year patency of graft in Cabrol-type anastomosis was 91.4% ± 1.2%, 88.8% ± 1.4%, 80.7% ± 2.2%, and 76.3% ± 3.7%, respectively.

## Discussion/Conclusion

This alternative proximal anastomosis technique in CABG demonstrated relatively comparable patency of aortocoronary graft.

